# Comparative analysis of adjuvant therapy for stage III BRAF‐mut melanoma: A real‐world retrospective study from single center in China

**DOI:** 10.1002/cam4.5866

**Published:** 2023-04-04

**Authors:** Jingqin Zhong, Wei Sun, Tu Hu, Chunmeng Wang, Wangjun Yan, Zhiguo Luo, Xin Liu, Yu Xu, Yong Chen

**Affiliations:** ^1^ Department of Musculoskeletal Surgery Fudan University Shanghai Cancer Center Shanghai China; ^2^ Department of Medical Oncology Fudan University Shanghai Cancer Center Shanghai China

**Keywords:** adjuvant therapy, BRAF mutation, Dabrafenib, melanoma, PD‐1 inhibitor, Trametinib, Vemurafenib

## Abstract

**Background:**

BRAF V600 mutation is the most common oncogenic alternation in melanoma and is visible in around 50% of cutaneous and 10%–15% of acral or mucosal subtypes. Currently, immunotherapy with anti‐PD‐1 blockade and dual‐targeted therapy with Dabrafenib plus trametinib (D + T) target therapy have been approved as adjuvant therapies for Stage III melanoma with BRAF V600 mutation. According to their phase III clinical trials, 3‐year recurrence‐free survival (RFS) is around 60% for both types of treatment. However, early disease control was slightly more effective with targeted therapy than immunotherapy. With different drug approval deadlines in China, anti‐PD1 monotherapy, D + T combination, and Vemurafenib (V) monotherapy have all been used in real clinical practice as adjuvant settings for stage III BRAF‐mut melanoma in recent years. We conducted this retrospective study to evaluate the efficacy of different treatments in the Chinese melanoma population.

**Methods:**

Patients who underwent radical surgery and were diagnosed as Stage III melanoma harboring BRAF V600 mutation by pathological report were retrospectively identified at Fudan University Shanghai Cancer Center from January 2017 to December 2021. Patients with mucosal melanoma, or with follow‐up of <6 months, or receiving other adjuvant treatment were excluded. Pearson's chi‐squared test or Fisher's exact test was performed for univariable analysis of the different adjuvant groups. Log‐rank analysis was used to identify prognostic factors for relapse‐free survival (RFS).

**Results:**

Ninety‐three patients with resected stage III melanoma with BRAF V600E mutation were identified in our study, including 25 patients receiving adjuvant anti‐PD‐1 immunotherapy (PD‐1), 25 receiving adjuvant D + T, 23 receiving V, and 20 patients with observation‐only (OBS). There were no statistical differences between treatment groups in baseline characteristics including age, gender, subtypes, primary thickness, ulceration, and nodal involvement. Median relapse‐free survival (RFS) time was not reached in the D + T group, 15 months in the V group, 15 months in the PD‐1 group, and 10 months in the OBS group, respectively. Compared to OBS, all three other groups showed a tendency to benefit from RFS, while only D + T achieved a statistical difference (*p* = 0.002). However, compared to D + T, anti‐PD‐1 monotherapy also showed significantly worse relapse control (*p* = 0.032).

**Conclusions:**

For Chinese stage III melanoma with BRAF mutation, both novel targeted therapy and immunotherapy showed potential benefits in relapse‐free survival compared to observation only. Dual‐targeted D + T therapy may still be the best choice for adjuvant therapy because anti‐PD‐1 monotherapy has failed to report equivalent efficacy in real‐world practice.

## Graphical abstract

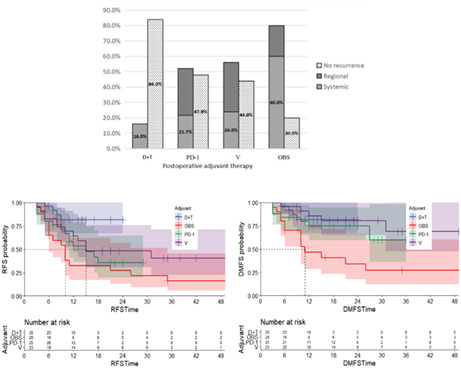

## 1 INTRODUCTION

Despite of the relatively much lower incidence rate of melanoma in Asian counties including China, it has shown a totally difference in predominate subtypes and genomic alternations here compared to the western world.^1^, ^2^ In the Caucasian population,^3^ mucosal melanoma, accounting for only 1.4% of all melanomas, is a rare subtype of melanoma with few adequate epidemiological research data on this subtype, whereas data from Asian countries such as China and Japan show that acral melanoma is the predominant subtype, accounting for 40%–50%, and mucosal types also account for 15%–20%. Nearly half of the cutaneous melanoma can harbor a single nucleotide variation in BRAF gene, mostly in V600 allete. However, less than 15% of acral or mucosal lesion harbor this type of mutation.^4^ Although melanoma with BRAF mutation is considered to be a more aggressive disease than its wide‐type, therapeutic approaches targeting BRAF V600E/K were the first and most successful precise treatment of melanoma so far. Combination of inhibitors for BRAF and MEK in MAPK pathway has become a standard front‐line therapy for BRAF‐mutant melanoma.^5^, ^6^

Patents with nodal involvement or in‐transit metastasis are still with high risk of recurrence after a radical resection. Therefore, 1‐year‐duration adjuvant therapy of novel immunotherapy or targeted therapy has been highly recommended in various clinical guideline for Stage III melanoma. According to the phase III trials Checkmate 238 and Keynote 054, both Pembrolizumab and Nivolumab can bring benefit for relapse‐free survival (RFS) and distant metastasis‐free survival (DMFS) in adjuvant setting.^7^, ^8^ In terms of adjuvant targeted therapy, Vemurafenib (V) monotherapy failed to improve post‐surgery outcomes in BRIM‐8 trial.^9^ Later COMBI‐AD trial demonstrated dual‐targeted therapy of Dabrafenib plus Trametinib (D + T) significantly reduced the risks of recurrence and metastasis for Stage III BRAF‐mutant melanoma.^10^

Due to the different time of drug approval of those anti‐PD1 blockades, BRAF and MEK inhibitors in China, we have treated Stage III melanoma in real‐world clinical practice, either with adjuvant immunotherapy using Pembrolizumab or other anti‐PD1 inhibitor, or with adjuvant Vemurafenib monotherapy or Dabrafenib plus Trametinib combination in the past. However, it remains controversial whether immunotherapy or targeted therapy can be more optimal for BRAF‐mutant melanoma. Therefore, we conducted this retrospective study to compare the efficacy of different adjuvant treatment for Stage III BRAF V600 mutant melanoma in our single centers.

## 2 MATERIALS AND METHODS

### 2.1 Patients

We retrospectively identified melanoma patients who received radical operation and diagnosed with Stage III disease and with BRAF V600 mutation by pathological assessment in Fudan University Shanghai Cancer Center (FUSCC) from January 2017 to December 2021. Patients with mucosal melanoma, or receiving adjuvant treatment other than anti‐PD monotherapy, V and D + T, or with follow‐up <6 months were excluded.

Pathological stage was determined according to the 8th edition of the American Joint Committee on Cancer (AJCC) cancer staging principle.^11^ BRAF mutations were detected by PCR, ARMS, or next‐generation sequency (NGS) from formalin‐fixed paraffin‐embedded (FFPE) tissue.

Clinicopathological characteristics and follow‐up information were retrieved from medical documents from our hospital database. All patients identified have signed an informed consent document during the preoperative conversation. This study was approved by the Medical Ethics Committee of FUSCC, and all methods were performed in accordance with the Declaration of Helsinki and the relevant guidelines and regulations.

### 2.2 Study treatment

All patients received a standard radical surgery including wide extended excision of primary lesion, sentinel lymph node biopsy (SNB) if with no evidence of clinical nodal involvement, and complete lymph node dissection (CLND) if with clinical positive nodal disease. CLND was not mandatory for patients with positive SN. Further decision on complete dissection was based on patients' own will and physician's recommendation.

All patients initiated their adjuvant treatment within 1 month after the surgery. Patients were categorized into four groups according to their postoperation therapy. Patients in PD‐1 group received Pembrolizumab 100 mg or 200 mg every 3 weeks, or Toripalimab 240 mg every 2 or 3 weeks. Toripalimab is another anti‐PD1 blockade developed by domestic pharmaceutical company and approved for melanoma in China. Patients in V group received oral Vemurafenib(V) 960 mg twice daily. Patients in D + T group received oral Dabrafenib 150 mg twice and Trametinib 2 mg once daily. All patients were scheduled to receive adjuvant therapy until disease‐relapse or at most 1 year. Decisions of dose reduction, treatment delay, and discontinuation were also made according to unacceptable adverse event and patients' tolerance. A group of patients with no further adjuvant treatment but observation (OBS) was also paired in our study.

### 2.3 Follow‐up

Patients were followed up with physical examinations and imagological assessment including ultrasound, CT, or MRI every 3 months for the first 2 years, every 6 months for 3–5 years, and then annually. Recurrence or metastasis was confirmed by pathologic or imaging test. Patients were followed up until death or May 30, 2022.

The regional relapse was defined as recurrence in primary lesion, original dissected or biopsied lymph node basin, or in‐transit disease. The systemic relapse was defined as distant metastasis. RFS was defined as the time interval from radical surgery to local recurrence or distant metastasis.

### 2.4 Statistical analysis

Pearson's chi‐squared test or Fisher's exact test was performed for univariable analysis of the different category groups. The RFS rates were evaluated using Kaplan–Meier curves, and the differences between the groups were tested using the log‐rank test. Differences with a *p* value < 0.05 (two‐sided) were considered statistically significant. All statistical analyses were performed using R software, version 4.2.0 (http://www.R‐project.org) and SPSS (version 22.0; SPSS Company) software.

## 3 RESULTS

### 3.1 Baseline characteristics

Nighty‐three patients were included in this study, including 25 patients in PD‐1 group, 23 patients in V group, 25 patients in D + T group, and 20 patients in OBS group. The clinicopathologic characteristics of all patients are listed in Table 1. Of total, there were 53 (57.0%) female and 40 (43.0%) male, 62 (66.7%) cutaneous, 27 (29.0%) acral, and 4 (4.3%) with unknown subtypes.

**TABLE 1:** Baseline patients' clinicopathologic characteristics.

	D + T (%) *n* = 25	V (%) *n* = 23	PD‐1 (%) *n* = 25	OBS *n* = 20	*p* value
Gender					0.622
Female	15 (60.0)	13 (56.5)	16 (64.0)	9 (45.0)	
Male	10 (40.0)	10 (43.5)	9 (36.0)	11 (55.0)	
Age (median)					0.796
<60 years	17 (68.0)	14 (60.9)	18 (72.0)	12 (60.0)	
>=60 years	8 (32.0)	9 (39.1)	7 (28.0)	8 (40.0)	
Subtype					0.100
Acral	8 (32.0)	2 (8.7)	8 (32.0)	9 (45.0)	
Cutaneous	17 (68.0)	20 (87.0)	16 (64.0)	9 (45.0)	
Unknown primary	0	1 (4.3)	1 (4.0)	2 (10.0)	
T stage					0.665
T1	1 (4.0)	0	0	0	
T2	3 (12.0)	4 (17.4)	6 (24.0)	2 (10.0)	
T3	12 (48.0)	9 (39.1)	5 (20.0)	5 (25.0)	
T4	7 (28.0)	7 (30.4)	11 (44.0)	9 (45.0)	
Tx	2 (8.0)	3 (12.0)	3 (12.0)	4 (20.0)	
Ulceration					0.252
No	6 (24.0)	11 (47.8)	10 (40.0)	3 (23.1)	
Yes	19 (76.0)	12 (52.2)	15 (60.0)	10 (76.9)	
Nodal Involvement					0.419
Micro‐metastasis	15 (60.0)	9 (39.1)	15 (60.0)	10 (50.0)	
Macro‐metastasis	10 (40.0)	14 (60.9)	10 (40.0)	10 (50.0)	
N stage					0.312
N1	6 (24.0)	9 (39.1)	10 (40.0)	6 (30.0)	
N2	10 (40.0)	4 (17.4)	7 (28.0)	10 (50.0)	
N3	9 (36.0)	10 (43.5)	8 (32.0)	4 (20.0)	
Stage III subgroup					0.860
IIIA	3 (12.0)	2 (8.7)	3 (12.0)	0	
IIIB	3 (12.0)	4 (17.4)	2 (8.0)	6 (30.0)	
IIIC	14 (56.0)	12 (52.2)	16 (64.0)	10 (50.0)	
IIID	3 (12.0)	3 (13.0)	2 (8.0)	2 (15.4)	
IIIx	2 (8.0)	2 (8.7)	2 (8.0)	2 (10.0)	
Relapse mode (initial)					0.000
Regional	0	7 (30.4)	8 (32.0)	4 (20.0)	
Systemic	4 (16.0)	5 (21.7)	6 (24.0)	12 (60.0)	
No relapse	21 (84.0)	11 (47.8)	11 (44.0)	4 (20.0)	

Median age was 60 years (range: 38–79 years). Median Breslow thickness was 3.21 mm, and ulceration rate was 60.2%. In terms of Stage III subgroup, there were 8 (8.6%) IIIA, 15 (16.1%) IIIB, 52 (55.9%) IIIC, and 10 (10.8%) IIID. All patients identified were detected with BRAF V600E mutation. Baseline factors were well‐balanced among each group.

### 3.2 Recurrence patterns

**FIGURE 1:**
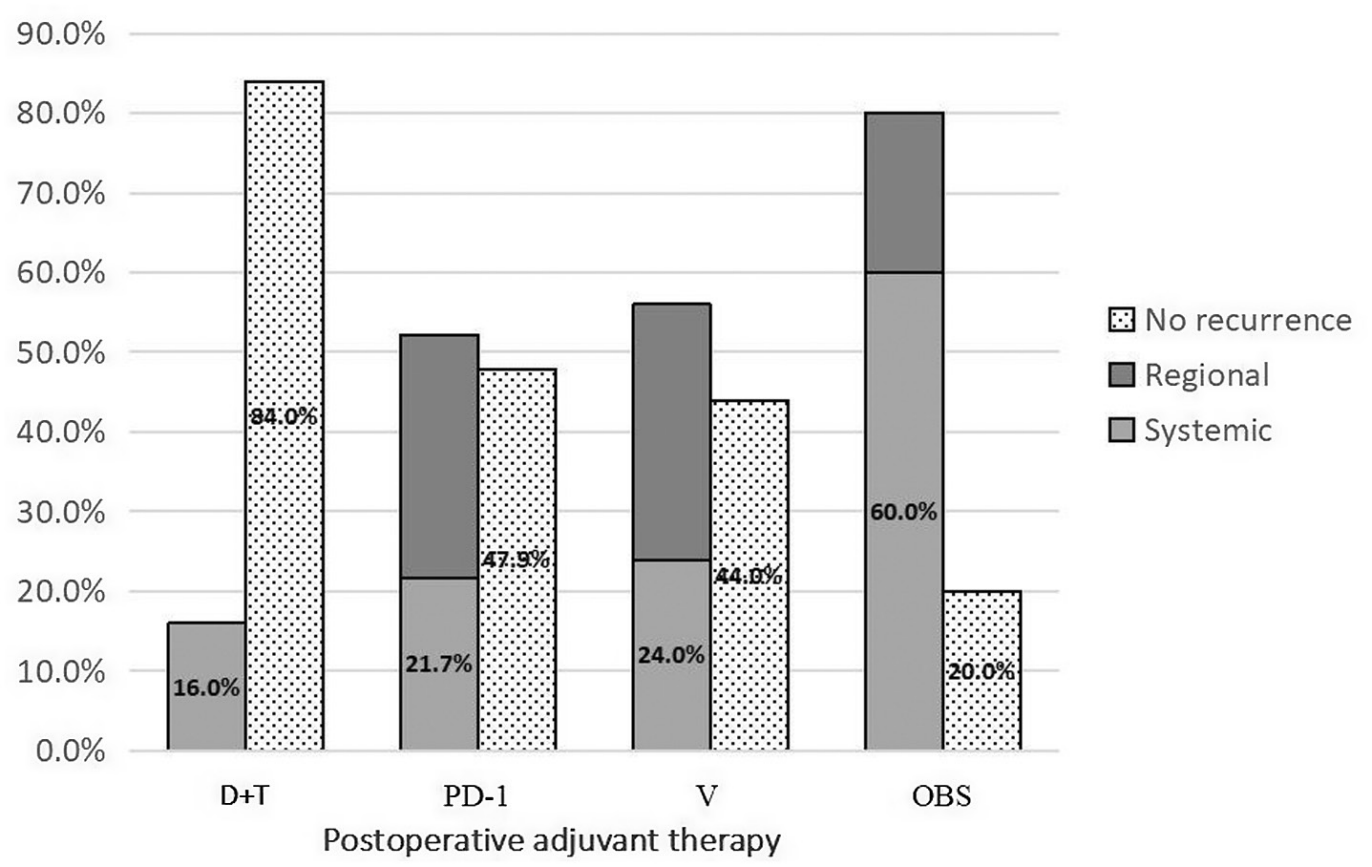
Proportion of recurrence patterns in each group after different postoperative adjuvant therapy.

Recurrence was detected in 46 (8.9%) of the 93 patients, with 4 (16.0%), 12 (52.1%), 14 (56.0%), and 16 patients (80.0%) in the D + T group, PD‐1 group, V group, and OBS group, respectively (Figure 1). Recurrence mode varied significantly among different group (*p* < 0.01). In the D + T group, the overall recurrence rate was approximately 16%, with 100% occurring systemic metastasis. Of these 14 (56.0%) recurrences in the V group, 8 (32.0%) were regional relapse, and 6 (24.0%) were systemic metastasis. In the PD‐1 group, the pattern of recurrence was slightly different, with 7 (30.4%) regional recurrences and 5 (21.7%) systemic relapses. With the highest recurrence rates (80.0%), over half (60.0%) of relapse in the OBS group were at distant sites. Therefore, among four groups, the patients in the D + T group had the lowest recurrence rate.

### 3.3 RFS analysis

Due to the different times of drug approval, median follow‐up time significantly varied among the four groups, with only 11 months in the D + T group, 35 months in the V group, 22 months in the anti‐PD‐1 group, and 48 months in the OBS group.

**FIGURE 2:**
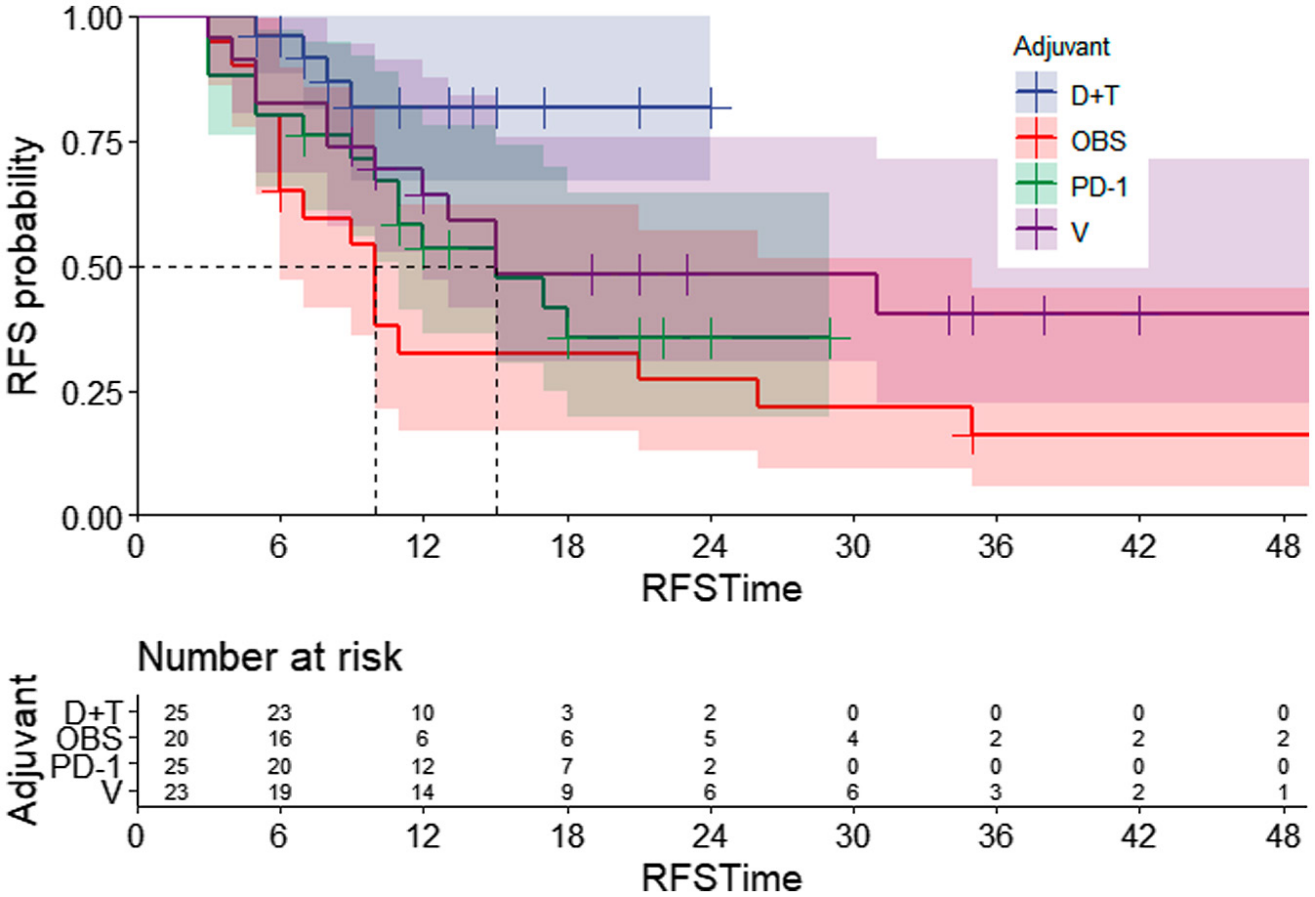
Recurrence‐free survival for all stage III melanoma patients stratified by postoperative adjuvant treatment. D + T, Dabrafenib plus Trametinib group; OBS, Observation group; PD‐1, Anti‐PD‐1 group; V, Vemurafenib group.

At the time of this report, the median RFS times of each group were, respectively, not reached in the D + T group, 15 months in the V group, 15 months in the anti‐PD‐1 group, and 10 months in the OBS group. The 1‐year and 2‐year RFS rates were 81.7% and 81.7%, 59.0% and 40.8%, 58.1% and 35.5%, 54.2% and 27.1%, respectively (Figure 2).

Compared with the OBS group, there were statistically significant benefits in RFS in all other groups (D + T vs. OBS, *p* = 0.003; V vs. OBS, *p* = 0.030; PD‐1 vs. OBS, *p* = 0.016). However, compared with the D + T group, both PD‐1 group (*p* = 0.032) and OBS group (*p* = 0.002) showed a worse survival, while the deteriorated outcomes in V monotherapy group did not reach statistical.

### 3.4 DMFS analysis

**FIGURE 3:**
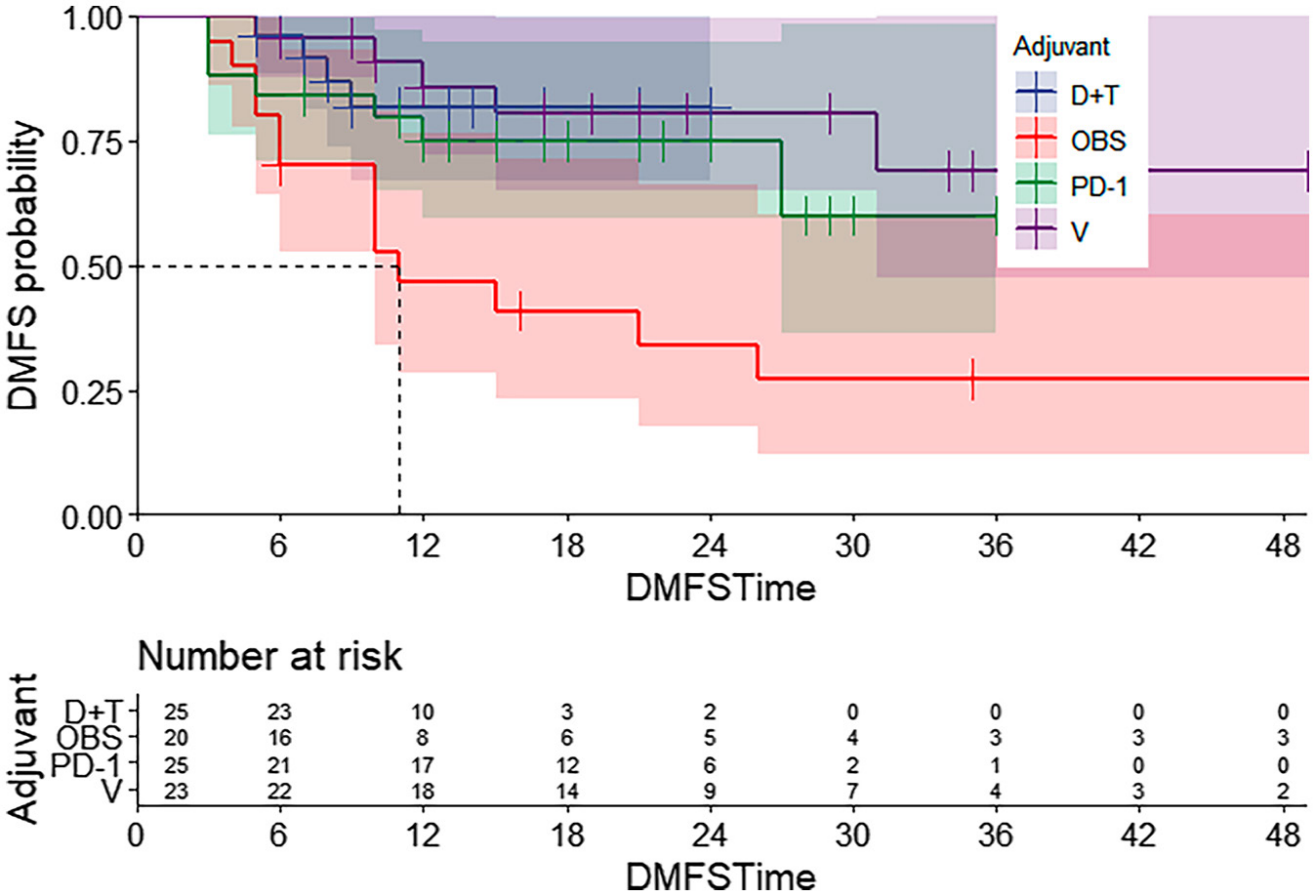
Distant metastasis‐free survival for all stage III melanoma patients stratified by postoperative adjuvant treatment. D + T, Dabrafenib plus Trametinib group; OBS, Observation group; PD‐1, Anti‐PD‐1 group; V, Vemurafenib group.

At the time of this report, the median DMFS times of each group were 11 months in the OBS group and not reached in the D + T group, the V group and anti‐PD‐1 group. Compared with the OBS group, there were statistically significant benefits in DMFS in all other groups (D + T vs. OBS, *p* = 0.0097; V vs. OBS, *p* = 0.0026; PD‐1 vs. OBS, *p* = 0.03; Figure 3). Although the adjuvant treatment groups showed any benefits in terms of distant metastases compared to the observation group, there was no statistical difference yet between the treatment modalities due to the short follow‐up period.

## 4 DISCUSSION

Our study is the first study to compare the efficacy of different adjuvant setting in Stage III BRAF‐mutant melanoma including one‐third of acral subtype in real‐world practice in Chinese population. In this retrospective study, relapse‐free survival benefits were observed in either anti‐ PD1 immunotherapy, mono or combination targeted therapy, compared to observation only. Additionally, regarding recurrence pattern or RFS analysis, Dabrafenib plus Trametinib combination brought the best survival outcomes.

In the first randomized phase III trial of adjuvant targeted therapy, Vemurafenib monotherapy failed to improve RFS of Stage IIIC (AJCC 7th edition) cohort and led to the overall negative result of BRIM 8 trial.^9^ Success of the following COMBI‐AD trial contributed to the only approval of adjuvant targeted therapy with Dabrafenib plus Trametinib combination for Stage III BRAF‐mutant melanoma.^10^ KEYNOTE 054 and CHECKMATE 238 also brought anti‐PD1 blockade immunotherapy to the adjuvant setting for both BRAF‐mutant and wide‐type melanoma.^7^, ^8^

However, arguments remain intensive about which treatment is better for patients with BRAF mutation disease in real‐world. According to the data from these clinical trials, both D + T combination and anti‐PD1 inhibitor can reduce nearly 40%–50% risk of both overall relapse and distant metastasis.^8^, ^9^, ^10^, ^12^ Patients receiving either type of adjuvant therapy reported RFS rate of nearly 60% at 3 years and 50% at 5 years.^8^, ^9^, ^10^ However, the median recurrence time was different among these two types of treatment. Among relapsed patients, 70% of cases receiving immunotherapy recurred within 1 year. The median recurrence‐free survival of anti‐PD1 therapy was about 5 months, which was recognized as the main reason of primary resistance.^13^ For adjuvant dual‐targeted therapy, nearly 80% recurrent cases relapsed when off the treatment and median recurrence time of D + T was around 18 months.^14^ Therefore, even though these two types of treatment might have similar long‐term outcomes, D + T combination seems to have better short‐term disease control than anti‐PD1 monotherapy in adjuvant setting.

Our study suggested that in real‐world experience, for Chinese melanoma, adjuvant immunotherapy did not show a comparable benefit to adjuvant dual‐targeted therapy, which was similar to some other studies. Recently in ASCO Annual meeting, a multicenter real‐world data of adjuvant treatment from German with 288 Stage III BRAF‐mutant melanoma patients identified, also showed that risk of recurrence was higher and earlier for immunotherapy than for targeted therapy.^15^ In their report, 1‐year and 2‐year RFS for adjuvant D + T was 88% and 66%, which was similar to the data from COMBI‐AD. But for anti‐PD1 immunotherapy, 2‐year RFS declined to only 45%, which was much worse than 68% reported in KEYNOTE 054. However, adjuvant anti‐PD1 monotherapy seemed to perform even worse in Chinese population in our study, with 1‐year and 2‐year RFS of only 58.1% and 35.5%. Considering the differences between Chinese and Western studies regarding the efficacy of immunotherapy, we think that BRAF‐mutant patients with acral subtype melanoma would have less sensitivity to immunotherapy because of the low levels of tumor infiltrating lymphocyte (TIL) in acral lentiginous melanoma^16^ and low mutational loads in BRAF‐mut melanoma.^17^

Comprised efficacy of immunotherapy in adjuvant setting for Asian population has been found aligned with the lower response for anti‐PD1 blockade in metastatic disease. In KEYNOTE 151 and Polaris‐01 trials, two trials of anti‐PD1 monotherapy conducted in Chinese melanoma patients, both Pembrolizumab and Toripalimab showed a much lower overall response rate (ORR) of only around 15% in acral or mucosal subtype.^18^, ^19^ In another international multicenter observational study with large samples of total 1135 patients receiving anti‐PD1 monotherapy, Caucasians had significantly higher ORR (54% vs. 20%) and longer progression‐free survival (PFS; 14.2 vs. 5.4 months) than East‐Asians/Hispanics/Africans even in non‐acral cutaneous and unknown‐primary population.^20^ These findings all suggested that our melanoma patients might be with more primary resistance to immunotherapy, due to worse immune microenvironment, less PD‐L1 expression, lower tumor mutation burden (TMB), and different drug resistance mechanisms.^21^, ^22^, ^23^ In CHECKMATE 238 Japan subgroup analysis, of total 28 patients identified including 4 acral and 3 mucosal cases, over 40% patients had PD‐L1 expression less than 1% and only around 25% had expression more than 5%. As it shown in result, 6‐month and 12‐month RFS in Nivolumab group was only 67% and 56%, much worse than the report of intension‐ to‐treat (ITT) population in the same trial.^24^ Additionally, there are a number of clinical studies on the dual drug immunotherapy. In the IMMUNED study,^25^ a phase II study of postoperative dual immunotherapy versus single anti‐PD1 and placebo in patients with stage IV melanoma, nivolumab (NIVO) + ipilimumab (IPI) adjuvant therapy was superior to NIVO in terms of RFS in patients with BRAF mutation subtypes. However, in another phase III adjuvant study (Checkmate915),^26^ NIVO + IPI did not show better outcomes than NIVO alone in Stage IIIB‐IV BRAF‐mut melanoma. Therefore, the efficacy of the combinatorial immune checkpoint blockade is still controversial. On the contrary, efficacy of targeted therapy might not deviate among different ethnicities. With long‐term follow‐up of the Chinese patients identified in multicenter phase IIa trial for Dabrafenib plus Trametinib conducted in East Asia, ORR reached as 71.7% and PFS was 9.3 months, which was comparable to the data from the clinical trials with Caucasian population.^27^

Our study still has several important limitations. First of all, it is a retrospective study with relatively small sample of patients in each treatment group. Exclusion of patients absent of intact medical data or adequate follow‐ups might lead to the bias in analysis and results. Due to the sequence of drug approval in China, D + T group had the shortest follow‐up of less than 1 year compared to other treatment. Whether the numeric benefit of dual‐targeted therapy can keep its significant superiority than anti‐PD1 or Vemurafenib monotherapy, requires longer follow‐up and larger patient cohort.

## 5 CONCLUSIONS

In conclusion, in real‐world clinical experience, anti‐PD1 inhibitor and D + T combination should be recommended for resected Stage III melanoma since they both improved RFS compared to only observation. However, dual‐targeted therapy of Dabrafenib plus Trametinib might be more optimal for BRAF‐mutant patients, since the relatively poor response to immunotherapy in Chinese population.

## AUTHOR CONTRIBUTIONS

**Jingqin Zhong:** Conceptualization (equal); software (equal); visualization (lead); writing – original draft (lead). **Wei Sun:** Data curation (equal); investigation (equal); resources (equal). **Tu Hu:** Data curation (equal); methodology (equal). **Chunmeng Wang:** Supervision (equal); validation (equal). **Wangjun Yan:** Supervision (equal); validation (equal). **ZhiGuo Luo:** Supervision (equal); validation (equal). **Xin Liu:** Supervision (equal); validation (equal). **Yu Xu:** Investigation (equal); methodology (equal); writing – review and editing (equal). **Yong Chen:** Data curation (equal); supervision (equal); validation (equal).

## FUNDING INFORMATION

This work was financially supported by the National Natural Science Foundation of China (Grant No. 82272857; 81802636), LinGang laboratory (Grant No. LG‐QS‐202205‐11).

## CONFLICT OF INTEREST STATEMENT

The authors made no disclosures.

## Data Availability

Not applicable.
